# Liposome and QS-21 Combined Adjuvant Induces theHumoral and Cellular Responses of Acellular Pertussis Vaccine in a Mice Model

**DOI:** 10.3390/vaccines11050914

**Published:** 2023-04-28

**Authors:** Baifeng Yang, Dewu Zhu, Yisi Zhou, Beizhe Gong, Yuan Hu, Jiayou Zhang, Shihe Huang, Xuanxuan Nian, Xinghang Li, Xinguo Li, Kai Duan, Xiaoming Yang

**Affiliations:** 1 National Engineering Technology Research Center for Combined Vaccines, Wuhan 430207, China; 2 Wuhan Institute of Biological Products Co., Ltd., Wuhan 430207, China; 3 National Biotec Group Company Limited, Beijing 100024, China

**Keywords:** *Bordetella pertussis*, vaccine, adjuvant, tissue-resident memory T cell, mouse model

## Abstract

The resurgence of pertussis in vaccinated communities may be related to the reduced long-term immunity induced by acellular pertussis vaccines. Therefore, developing improved pertussis vaccine candidates that could induce strong Th1 or Th17 cellular immunity is an urgent need. The use of new adjuvants may well meet this requirement. In this research, we developed a novel adjuvant candidate by combining liposome and QS-21 adjuvant. Adjuvant activity, protective efficacy, the level of neutralizing antibody against PT, and the resident memory T (T_RM_) cells in lung tissue after vaccination were studied. We then performed *B. pertussis* respiratory challenge in mice after they received vaccination with traditional aluminum hydroxide and the novel adjuvant combination. Results showed that the liposome + QS-21 adjuvant group had a rapid antibody and higher antibody (PT, FHA, Fim) level, induced anti-PT neutralizing antibody and recruited more IL-17A-secreting CD4^+^ T_RM_ cells along with IL-17A-secreting CD8^+^ T_RM_ cells in mice, which provided robust protection against *B. pertussis* infection. These results provide a key basis for liposome + QS-21 adjuvant as a promising adjuvant candidate for developing an acellular pertussis vaccine that elicits protective immunity against pertussis.

## 1. Introduction

Pertussis, also known as whooping cough or 100-day cough, is a highly contagious respiratory disease caused by Gram-negative *Bordetella pertussis* (*B. pertussis*). The disease was globally prevalent and has been a global health burden since a century ago [1]. The resurgence of the disease has prompted research and control measures, including the development of vaccines. Currently, whole-cell pertussis vaccines (wP, first generation) and acellular pertussis vaccines (aP, second generation) are the only licensed pertussis vaccines. Previous use of wP vaccines has led to a reduction in the incidence of pertussis. However, due to safety concerns of wP, such as the reactogenicity and side effects, the aP vaccine replaced the wP vaccines in many countries during the 1990s and 2000s. For example, in China, wP was replaced by aP in 2008. However, in recent years, periodic outbreaks of the disease have been reported in adolescents or adults, even in vaccinated populations, for example, in USA [2], Japan [3], Australia [4], and China [5]. The resurgence of pertussis has been attributed to several factors. The main reason may be the weakened duration of the immune response induced by aP, coupled with pathogen adaptation due to changes in bacteria genome [6]. For instance, the emergence of *B. pertussis* mutant strains in pertussis toxin (PT) and pertactin (PRN) may escape the immune protection provided by current vaccines [7,8]. PT is one of the major virulence factors during *B. pertussis* infection, which plays a key role in pathogenesis. It is a hexamer composed of S1, S2, S3, two S4, and S5 subunits, in which S1 subunit contains the binding and catalytic sites. Additionally, PT is the most important antigen in aP and is responsible for vaccine reactogenicity.

The immune response induced by wP is similar to that induced by *B. pertussis* natural infection, mainly Th1- and Th17-type responses, which plays an important role in fighting against pertussis infection, while aP, as the component antigen, mainly induces Th2 and Th17-type responses [9,10,11]. Studies on animal models, including baboon models, have suggested that vaccination with aP could not prevent nasal colonization and transmission of *B. pertussis* [12]. Memory T cells, also known as tissue-resident memory T cells (T_RM_ cells), have been reported to play a crucial role in mucosal tissue during and after viral or bacterial infection, inducing a rapid immune response against pathogen re-infection [13]. Wilk et al. revealed that protective immunity induced by natural infection of *B. pertussis* and wP vaccination was more effective than that induced by aP vaccination. Protective immunity conferred by wP is mediated by respiratory T_RM_ cells [14].

All current aP vaccines are prepared by adsorbing *B. pertussis* antigens (PT, filamentous hemagglutinin (FHA), PRN, and fimbriae type 2 and type 3 (Fim2/3)) to aluminum salt. aP vaccines appear to confer adequate protection against clinical disease but are less efficient in preventing infection and, consequently, transmission [15]. As indicated before, the potential origin of the resurgence of pertussis is multifactorial. A deficient protective immune response induced by aP vaccines is one of the causes [16]. Recent research in animal models has revealed that experimental aP with novel adjuvants that elicit predominantly Th1-skewed responses provides better protection against *B. pertussis* infection than alum-adjuvant vaccines [10]. Such research findings suggest that modification to the current formulations may improve vaccine efficacy. Queenan et al. demonstrated that increasing the quantity of Fim proteins in pertussis vaccine formulations may enhance the efficacy of the vaccine against pertussis without increasing the reactogenicity of the vaccine [17].

Apart from modifying or adding vaccine antigens, vaccines might also be improved by including new adjuvants [18]. In addition to the aluminum salt adjuvant, new adjuvants such as AS03, MF59, AS04, AS01 and CpG have been approved for human use in vaccines [19]. Liposomes are characterized by a lipid bilayer structure, which serves as an adjuvant and a carrier concurrently. Owing to their structural properties, liposomes are completely biodegradable and can encapsulate both hydrophilic and lipophilic antigens. After administration, antigens encapsulated by liposomes can avoid degradation and thereby release slowly and constantly, stimulating prolonged immune responses [19]. QS-21 is a saponin adjuvant purified from the bark of the Chilean tree, *Quillaja saponaria*. It stimulates the production of NLRP3 inflammasome, causing the release of IL-1b and IL-18, promotes cytotoxic T-lymphocytes (CTLs) production, and generates Th1 cytokines, including IL-2 and IFN-γ [19]. QS-21, as part of AS01B adjuvant, was approved by FDA for vaccines for human use [19]. Many studies have demonstrated that these new adjuvants not only increase humoral immunity, but also induce augment cellular immunity.

All this information, along with the latest knowledge of antigenicity of circulating *B. pertussis* strains, forms the basis for potential strategies to develop new and improved pertussis vaccines. In this study, we designed a reasonable vaccine formula containing liposome and QS-21, to ensure vaccine efficacy and guarantee the compatibility of pertussis antigens with the adjuvants. The immune response of the formulations was evaluated in the mouse model.

## 2. Materials and Methods

### 2.1. Ethics Statement

This study protocol was approved by the Animal Ethics Committee of the Wuhan Institute of Biological Products (WIBP) (WIBP-AII no. 362020001). All experiments were performed following the relevant guidelines and regulations in China [20].

### 2.2. The Preparation of aP Vaccine

All reference antigens used were provided by WIBP. All antigens used for aP vaccine preparation were purified from *B. pertussis* cell lysates. The clinical-grade antigen purified PT (which is glutaraldehyde detoxified), FHA, PRN, and fimbriae type 2 and type 3 (Fim2/3) were detected by SDS-PAGE according to the previously reported method [17]. The endotoxin contents of antigens and adjuvants were determined before vaccine preparation. A total of 50 μg dPT, 50 μg FHA, 16 μg PRN and 10 μg Fim2/3 were determined in aP. For the traditional aP (AL-aP), the standard content antigen was adsorbed onto aluminum hydroxide (alum) suspension (WIBP) (0.49 mg/mL Al^3+^) into a final volume of 1 mL. For the new combined adjuvant aP vaccine (LQ-aP), standard content antigen was combined with 1.07 mg liposome (Maximmune, Chengdu, China) and 100 μg QS-21(Maximmune, Chendgu, China). The size distribution and Zeta potential of the adjuvants and the two formulated vaccines were examined by Laser Particle Sizer (EPA2000, Malvern) and Zetasizer (ZS90, Malvern). wP vaccines were obtained from WIBP.

### 2.3. Mouse Immunization

For the immunogenicity study, CD1 mice (female, 20–24 g) and C57BL/6 mice (female, 6–8 weeks old) were purchased from Vital River Laboratory Animal Technology Co., Ltd., Beijing, China; CD1 mice were divided into 3 groups. Each group was intraperitoneally immunized once with 100 μL vaccines of AL-aP, LQ-aP, and normal saline (NS). Sera for pertussis antigen-specific IgG antibodies and PT neutralization antibody detection were collected on the 7th, 28th, 35th, and 42nd day after immunization.

For *B. pertussis* challenge study, C57BL/6 mice were divided into 4 groups, each group immunized i.p. with 100 μL of vaccines of AL-aP, LQ-aP, wP, and NS twice at an interval of 4 weeks and intra-nasally challenged with a virulent strain of *B. pertussis* (BpCMCC58003; 1 × 10^9^ CFU/mL) as described previously [21] 1 week later. Sera were collected before the challenge for antibody subtyping detection, and spleen was collected before the challenge and days 3, 5, and 10 post-challenge, respectively. Bacterial burden at different time intervals post-infection was evaluated by performing CFU counts on serially diluted lung homogenates from individual mice. The wP vaccines were used as positive control, whereas normal saline (NS) was used as a negative control.

### 2.4. Serological Assays

#### 2.4.1. Pertussis Antigen-Specific Antibodies Titer Detected by ELISA

Pertussis antigen-specific antibodies were quantified by ELISA with diluted sera. Briefly, the purified PT, FHA, PRN, and Fim2/3 antigens were diluted to 5 μg/mL. The contents were then separately added to microtitration plates (Greiner bio-one, Frickenhausen, Germany) and reacted overnight at 4 °C with carbonate coating buffer. The plates were washed four times with washing buffer (PBS buffer containing 0.05% Tween 20). For blocking, 100 μL of blocking buffer (1% bovine serum albumin in PBS) was added to each well and incubated for one hour at 37 °C. Then, the plate was washed 3 times with a washing buffer. *B. pertussis* anti-serum (mouse) (97/642, WHO Reference Reagent, NIBSC) and serum samples diluted to a specific concentration (1:200, days 7; 1:800, days 28, 35, and 42) were added to the wells. This was then followed by the addition of a pre-prepared KPL Peroxidase-Labeled Antibody (Sera Care, Milford, MA, USA). The absorbance of each well was read using a SpectraMax^®^ ABS Plusplate reader (Molecular Devices) at 405 nm. Using the method described previously [22], ELISA concentration units were calculated from mouse reference 97/642 to PT of 17 units, FHA of 143 units, PRN of 30 units, and Fim2/3 of 32 units. Antibody levels are expressed as the Geometric mean titer (GMT).

#### 2.4.2. PT Neutralization Assay

The PT-functional IgG-titers were measured on day 24 after immunization. The PT neutralization assay was carried out in line with principles described previously with slight modification [23]. Briefly, two-fold serially diluted sera (CD1) were mixed with 4 CTU_100_ (the minimal dose of active PT needed to cause 100% cell clustering in 2 h) of JNIH-5 (2.5 ng/mL) (NIBSC) and incubated for 2 h at 37 °C. A total of 50 μL of serum/PT mixtures was added to CHO-K1 cells (3 × 10^4^ cells/well) (National Institutes for Food and Drug Control, China) incubated for 48 h at 37 °C. Cells were stained with crystal violet and evaluated for morphological alterations (clustered phenotype) by light microscopy. Endpoint titers are the reciprocal of the highest dilution able to inhibit cell clustering. The serum of rats immunized with Infanrix^®^ (London, UK, GSK) was used as a positive control.

#### 2.4.3. IgG Subtyping

Mouse IgG2c ELISA Kit (Bethyl, Montgomerie, NY, USA) and IgG1 ELISA Kit (Bethyl, Montgomerie, NY, USA) were used for C57BL/6 serum IgG subtyping.

### 2.5. Detection of Respiratory Tissue-Resident T Cells

We used a well-described approach to discriminate tissue-resident from circulating CD4 T cells [24]. Briefly, C57BL/6 mice immunized with different vaccines were injected with an anti-mouse FITC-CD45 Ab (Bio Legend, Santiago, MN, USA) intravenously (i.v.) 10 min before euthanasia. Circulating lymphocytes were exposed to the antibody and labeled CD45iv^+^, whereas tissue-resident cells were “protected” and remained CD45iv^−^. “Tissue-resident CD4^+^ T cells” were defined through the expression of CD4 and lack of in vivo labeling of CD45 after i.v. injection of mice with anti-CD45 10 min before euthanasia.

### 2.6. Flow Cytometry Analysis

Lung tissue was prepared mechanically, followed by enzymatic disruption of tissue for 1 h at 37 °C with Collagenase (2 mg/mL; Diamond) and DNase I (10 U/mL; Sigma, St. Louis, MO, USA) [14]. Spleens were grinded. Next, lungs and spleens were passed through a 70 μm cell strainer to obtain a single cell suspension, followed by RBC lysis. The cells were then incubated with CD16/CD32 (FcγRⅢ/Ⅱ, BD Biosciences, San Jose, CA, USA) to block IgG Fc receptors. Cells were incubated with LIVE/DEAD^®^ Aqua (Invitrogen, Waltham, MA, USA), followed by surface staining with fluorochrome-conjugated anti-mouse Abs for various markers. To detect cytokines, cells were stimulated with Cell Activation Cocktail (with Brefeldin A, Biolegend, San Diego, CA, USA) for 5 h at 37 °C. The cells were then fixed, permeabilized, and stained with IL-17A-APC, IFN-γ-PE, and IL-4-BV421 (Biolegend). The following antibodies to cell surface markers were used: CD45-FITC, CD3- APC/Cyanine7, CD4-PE-Cyanine7, CD8a-PerCP/Cyanine5.5, CD44-BV650, CD62L-APC-eFluor780, CD69-BV605, and CD103-Alexa Fluor700. Flow cytometry analysis was performed on a CytoFLEX S, and data were acquired using CytExpert software (Beckman). The results were also analyzed using CytExpert software (Beckman). Tissue-resident T cells (defined through lack of in vivo labeling with anti-CD45 as described above) that were CD44^+^ CD62L^-^ and expressed CD69, with or without CD103, were considered “tissue-resident memory T cells”.

### 2.7. Statistical Analysis

Statistical analysis was carried out using Graph Pad Prism 8.0 software (Graph Pad Software Inc., San Diego, CA, USA). One- or two-way analysis of variance (ANOVA) followed by Tukey’s multiple comparison tests was used to analyze statistical significance among three or more groups. A *p* value of < 0.05 was considered statistically significant. Data from animal studies were compiled in Microsoft Excel.

## 3. Results

### 3.1. Pertussis Antigen and Adjuvant Formulations Characterization

**Figure 1:**
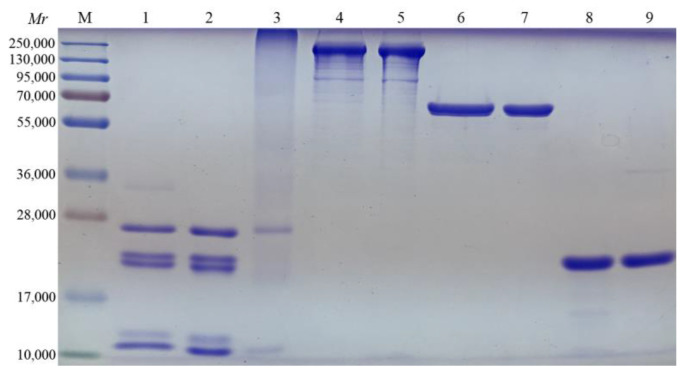
SDS-PAGE (5–12%) stained with Coomassie blue R250. *Mr*, molecular weight. M, protein marker (26616#, Thermo Fisher, Waltham, MA, USA). 1, PT reference. 2, PT not detoxified. 3, PT detoxified. 4, FHA reference. 5, FHA. 6, PRN reference. 7, PRN. 8, Fim2/3 reference. 9, Fim2/3.

After purifying antigens from B. pertussis cell lysates, the identities of component pertussis antigens were confirmed using SDS-PAGE. Purified PT contains five subunits S1, S2, S3, S4, and S5, with complete structure. After detoxification, PT subunits polymerized and diffused to the high molecular weight area, increasing molecular weight. The FHA showed four bands in which the molecular weight of the main band was 220,000, accounting for more than 75% of the total weight. The molecular weight of PRN was 69,000, while that of Fim2/3 was between 22,000 and 22,500. The purity of antigen of all components was more than 95%, which meets the requirements of vaccine preparation (Figure 1).

Vaccines were prepared and characterized to guarantee adequate formulation quality. There was no significant change in particle size before and after antigen addition in combination LQ adjuvant (Table 1). Zeta potential was negative, which was consistent with the negative charge of liposome at pH 7.0 [25]. In the Alum adjuvant group, the average particle size of the vaccine after adsorption (6.16 μm) was larger than that of the Alum adjuvant (2.61 μm). On the other hand, the zeta potential after antigen adsorption (10.03 mV) was smaller than that of Alum (22.53 mV). The protein content in the supernatant of the vaccine as detected by Lowry method indicated that adsorption of pertussis antigens onto Alum was 97%, which met the requirement of pharmacopoeia for adsorption rate ≥ 95% (Table 1) [26].

### 3.2. Serum IgG Responses to Pertussis Vaccines

**Figure 2:**
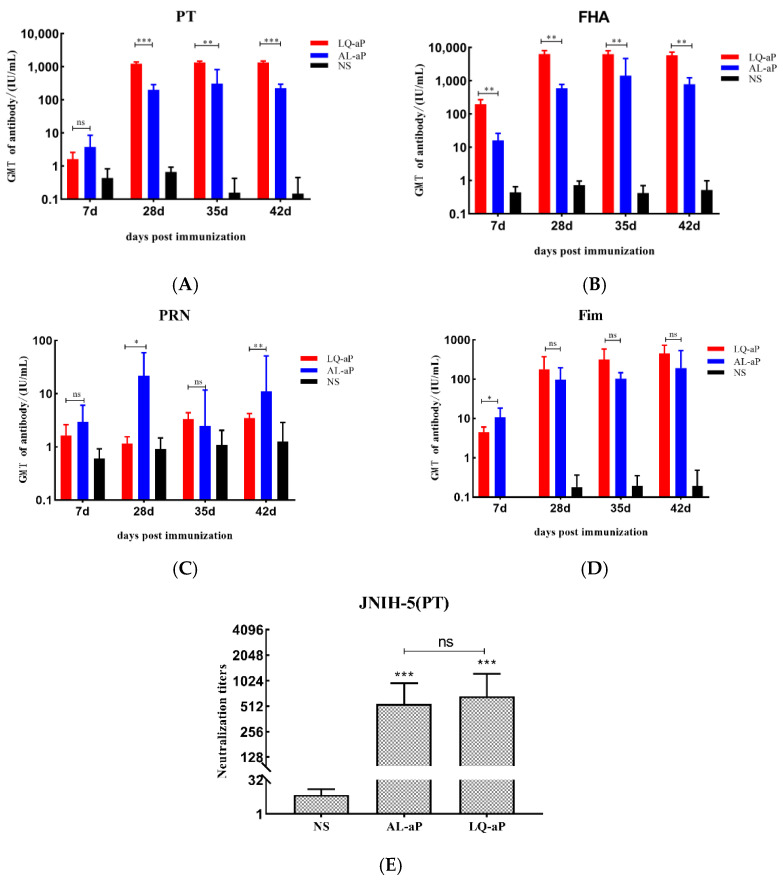
The results of the serological test in CD1 mice. CD1 mice received intraperitoneal injection (i.p.) of 100 μL different aP formulations. Serum antibody titers anti-PT (**A**), anti-FHA (**B**), anti-PRN (**C**), and anti-Fim2/3 (**D**), after 7, 21, 35 and 42 days of immunizations with vaccine were detected using ELISA. The red, blue, and black columns represent LQ-aP, AL-aP, and NS groups, respectively. Results are geometric mean ± SD of mice per group per time point. (**E**) Day 42 in vitro neutralization titers of anti-PT (JNIH-5) using different sera. Results are geometric mean ± SD mice per group. ns: non-significant, * *p* < 0.05, ** *p* < 0.01, *** *p* < 0.001 by two-way ANOVA with the Tukey’s post hoc test.

The analysis of pertussis antigen-specific serum IgG indicated that the use of LQ and Alum adjuvants in aP vaccine formulations elicited potent humoral immune responses (Figure 2). When pertussis antigens were administered, the level of serum anti-pertussis IgG antibody against PT, FHA, PRN, and Fim2/3 antibodies was readily detected on day 7 after immunization and increased gradually, and a plateau period was reached on day 28.

The antibody against PT and FHA induced of LQ-aP group was significantly higher than that of the Alum group (Figure 2A,B). Anti-PT and anti-FHA IgG of the AL-aP group increased significantly and reached the highest level in 35 days and declined in 42 days (Figure 2B). The anti-PRN IgG level in the Alum group was better than other adjuvant groups on the 7th and 28th day (Figure 2C). Although there was no statistical difference in the level of anti-Fim IgG level between the LQ-aP and AL-aP groups, from the 28th day, the level of anti-Fim IgG level in the LQ-aP group was generally higher than that in the AL-aP group (Figure 2D).

To evaluate the ability of the induced antibodies to recognize antigenic functional epitopes, pertussis toxin neutralization assays were performed (Figure 2E). We determined a significant difference in neutralizing antibody level between the NS group and LQ-aP or AL-aP groups *(p* < 0.001), but no significant difference was detected between adjuvant groups (*p* > 0.05).

### 3.3. The Proportion of CD4^+^ Cells in T Lymphocytes after LQ-aP Immunization

**Figure 3:**
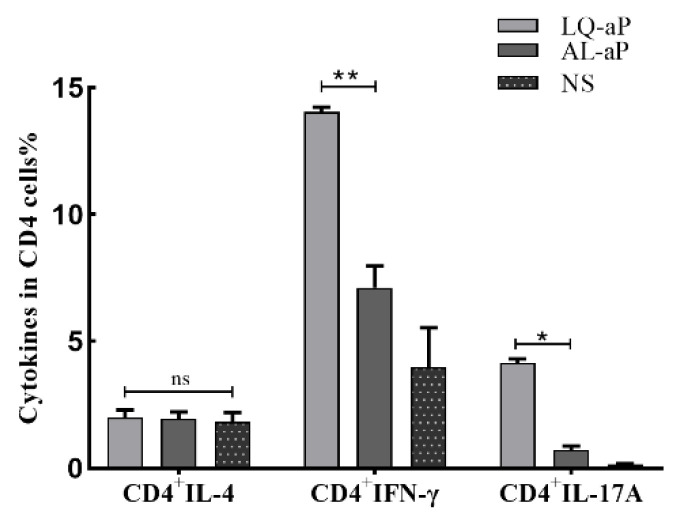
The proportion of IFN-γ-, IL-4-, and IL-17A-secreting CD4^+^ T cells in the spleens with flow cytometry analysis. The results shown are mean ± SD. ns: non-significant, * *p* < 0.05, ** *p* < 0.01, by two-way ANOVA with the Tukey’s post hoc test.

To estimate the level of cellular and humoral immunity induced by LQ-aP and AL-aP, the vaccine-specific spleen lymphocytes of C57BL/6 mice induced by LQ-ap and AL-aP were detected by flow cytometry. IL-4 mainly contributes to humoral immunity, while IFN-γand IL-17A primarily account for cellular immunity. Compared with other immunized groups, the proportion of CD4^+^ T cells in the NS group was lower. In vitro stimulation and antibody labeling of CD4^+^ T cells secreting different cytokines (IFN-γ-, IL-4-, and IL-17A), the proportion of IFN-γ-secreting and IL-17A secreting CD4^+^ T cells in the LQ-aP group were higher than those proportions in AL-aP group (*p* < 0.05) (Figure 3). No significance was observed in the level of IL-4 secreting CD4^+^ T cells (Figure 3), which is consistent with the observation from Figure 2E that neutralizing antibody titers were similar in both adjuvant groups. These results exhibit the involvement of both humoral and cellular immunity in the LQ-aP vaccine group.

### 3.4. CD4 ^+^T_RM_ Cells Induced in the Lung Tissue by the LQ-ap Vaccination Group Potently Secrete IFN-γ

**Figure 4:**
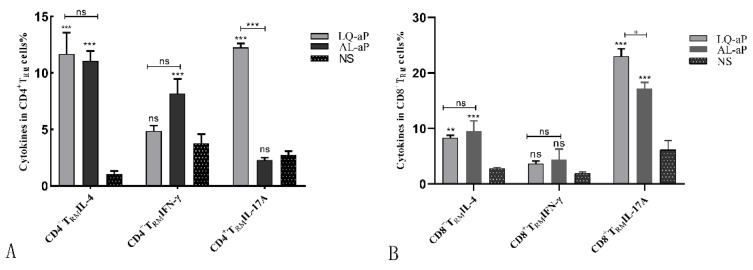
(**A**): The proportion of IFN-γ-, IL-4-, and IL-17A-secreting CD45^iv−^CD44^+^CD62L^−^CD69^+^CD103^±^CD4 T_RM_ cells in lungs after immunization. (**B**): The proportion of IFN-γ-, IL-4-, and IL-17A-secreting CD45^iv−^CD44^+^CD62L^−^CD69^+^CD103^±^CD8 T_RM_ cells in lungs after immunization. The results shown are mean ± SD. ns: non-significant, * *p* < 0.05, ** *p* < 0.01, *** *p* < 0.001 by two-way ANOVA with the Tukey’s post hoc test.

We performed intracellular staining (ICS) and flow cytometry on the cells isolated from the lung tissues of mice immunized with LQ-aP and AL-aP vaccine groups. Intravenous staining of circulating leukocytes was performed to discriminate circulating (CD45^iv+^) from tissue-resident (CD45^iv−^) CD4 T cells. In this CD45^iv−^ population, CD44^+^CD62L^−^ cells and CD69-expressed cells were defined as T_RM_ cells. In contrast with the NS group, the LQ-aP group induced enhanced production of IL-4, IFN-γ, and IL-17A secreting CD4^+^ cells, while the AL-aP group induced improved IL-4 and IFN-γ secreting CD4^+^ cell level (Figure 4A). For CD8^+^ T_RM_ cell response, AL-aP and LQ-aP enhanced IL-4 and IL-17 A secreting CD8^+^ T_RM_ response, while no significant difference was determined for IFN-γ secreting CD8 T_RM_ response among the three groups (Figure 4B).

### 3.5. Shift in the IgG1/IgG2c Profile

**Figure 5:**
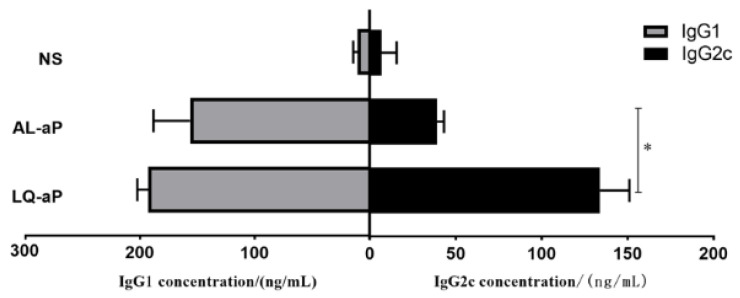
The antibody titers of IgG1 and IgG2c in C57BL/6 mice after two immunizations. The results shown are mean ± SD. * *p* < 0.05 by one-way ANOVA with the Tukey’s post hoc test. Only significant differences between experimental groups are indicated.

In order to access the balance of humoral and cellular immunity induced by the LQ-aP and AL-aP group, we examined the IgG1/IgG2c level using serum 7 days after the second immunization. Though a higher IgG1 antibody level was determined, there was a nonsignificant difference in IgG1 antibody between LQ-aP and AL-aP groups (Figure 5). As expected, compared with the AL-aP group, the LQ-aP vaccinated group showed significantly higher IgG2c concentration (Figure 5), indicating LQ-aP induces strong cellular and humoral immunity.

### 3.6. Results of Intranasal Challenge Protection against Pertussis in Mice

**Figure 6:**
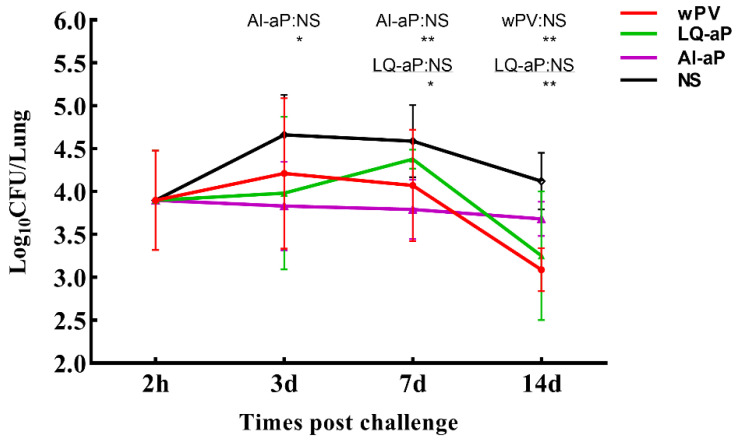
CFUs in the lungs were enumerated on days 3, 7, and 14 post-challenges. The results shown are mean ± SD of mice (n = 3). The results shown are mean ± SD. * *p* < 0.05, ** *p* < 0.01 by one-way ANOVA with the Tukey’s post hoc test. Only significant differences between experimental groups are indicated.

We investigated the potential of immune responses induced by LQ-aP and AL-aP vaccines to effectively prevent the colonization of pertussis in the lungs of C57BL/6 mice. Mice were challenged by exposure to *B. pertussis* 7 days after the second immunization. CFUs in the lung of mice in the NS group increased in the first 3 days and reached the peak on day 3. However, LQ-aP and AL-aP groups showed no increment in CFU in lung tissue before day 3. Importantly, there was a significant difference between AL-aP and NS groups on day 3. On day 7, although CFU in the NS group was lower than on day 3, it was still significantly higher than CFU in LQ-aP and AL-aP groups. On day 14, bacteria load was the lowest in all groups tested, and significant differences were observed between the LQ-aP group and NS group, as well as the wPV group and NS group (Figure 6). As a result, LQ-aP vaccination could effectively protect mice from *B. pertussis* infection.

## 4. Discussion

In this study, we developed acellular pertussis vaccines using liposome + QS-21 adjuvant and compared this novel adjuvant combination with traditional aluminum hydroxide adjuvant by studying antigen-specific antibodies, PT-neutralizing antibodies, antibody subtypes, and tissue-resident memory T cell levels. The *B. pertussis* respiratory challenge in mice of LQ-aP shows higher viral clearance than the AL-aP group. The significant production of IgG2c in the LQ-aP group demonstrates the involvement of cellular immunity. Additionally, we determined that liposome + QS-21 adjuvant induces humoral immunity, as well as IL-17-secreting T_RM_ cells that account for cellular immunity, indicating comprehensive immune protection. Considering all these results, we conclude that the liposome + QS-21 adjuvant is a promising adjuvant candidate for the acellular pertussis vaccine.

Although the vaccination rate of pertussis vaccines is very high in many countries, including China, more and more cases of pertussis have been reported in recent years. Some experts call this increase in pertussis cases “re-emerging pertussis” [27,28]. By comparing the humoral and cellular immunity induced by aP vaccine, wP vaccine, and pertussis natural infection, it was determined that one of the main reasons for this phenomenon is the humoral immunity and weakened immunity induced by aP vaccine [29]. There is increasing evidence from animal models that the immunity generated with aP vaccines does not prevent nasal mucosal infection [14]. Pertussis vaccine antigens were purified separately. The standard purity required (purity > 95%) for vaccine development was attained. The PT antigen was treated with glutaraldehyde and applied to different pertussis vaccines. The size distribution and zeta potential pre- and post-formulation were unchanged, revealing that pertussis antigens remained free in the aqueous phase of the liposome. These findings are similar to those of a study carried out by Brito [30]. The antigen adsorption on the surface of the adjuvant is an important parameter of vaccine quality [31]. Our results indicate that the adsorption of antigens in AL-aP was more than 95%, which meets the requirement of the Chinese pertussis vaccine standard [26]. The decrease in zeta potential after adsorption suggested that pertussis antigens were adsorbed on the surface of the adjuvant, and this ensured the stability of the antigen in the aluminum adjuvant vaccine.

Serum tests showed that the new adjuvant increased the antibody titer of anti-PT, FHA, and Fim2/3 IgG. This implied that the new adjuvant could induce a stronger humoral immune response. There was no significant difference in anti-PRN antibody levels among adjuvant groups, and the anti-PRN antibody in all groups was lower compared with anti-PT, FHA, and Fim2/3 IgG, which has been observed in Seung’s study [32]. There are possible reasons for the low titer of anti-PRN IgG. First, the immunogenicity of the PRN antigen was influenced during purification and external conditions in various laboratories. Second, experiment mice were injected with a single dose. The higher IgG titers could be addressed after booster vaccination. The potency of the acellular pertussis vaccine was tested by a modified intra-cerebral challenge assay (MICA). The potency of Alum, LQ-aP vaccine groups reached that of the Chinese pertussis vaccine standard (≥8 IU/mL, data not published) [26].

To evaluate the immune response induced by the LQ+aP adjuvant vaccine, we carried out antibody subtyping and PT toxin neutralization test in vitro. The neutralizing antibody titers of AL-aP and LQ-aP group were similar, indicating that LQ-aP could also induce a better humoral immune response. In the literature, IgG2a/Th1 responses are associated with an efficient and rapid clearance of *B. pertussis* [10]. Importantly, the C57BL/6 mice produce the IgG2c subtype, which is an isomer of IgG2a. The concentration of IgG2c induced by the LQ-aP group was significantly higher than that of AL-aP, indicating that the LQ-aP group stimulates stronger cellular immunity. All in all, immunity generated by the LQ-aP group is more comprehensive than that of the AL-aP group, demonstrating wider protection against *B. pertussis*.

A mouse respiratory challenge model has been proven to be very useful for studying the mechanisms of protective immunity to *B. pertussis* [33]. The results of CFU counts showed that the wP vaccine might be fit to effectively prevent the colonization of pertussis in the respiratory tract, which was similar to the study by Wilk et al. [14]. LQ-aP was also effective in preventing bacterial colonization of pertussis in the respiratory tract, which is probably due to the cellular immunity induced by LQ-aP.

Given the importance of cell-mediated immunity in disease protection at the population level, novel vaccine strategies that target the appropriate immune responses are needed. In this study, we used liposome + QS-21 adjuvants to prepare the pertussis vaccine and evaluate the immune effect in a mouse model to balance cellular and humoral immunity. There is also a mainstream trend of adjuvant combinations such as AS04 developed by GSK company based on the aluminum adjuvant platform [34].

The T_RM_ cells have been reported to effectively prevent the spread of bacteria, viruses, or other pathogens [35]. Through analyzing lung tissue-resident memory T cells before the challenge by flow cytometry, we determined that LQ-aP not only induced IL-17A-secreting CD4^+^ T_RM_ cells, which was consistent with the results of other studies, but also detected IL-17A- secreting CD8^+^ T_RM_ cells [36]. The IL-17A-secreting CD8^+^ T_RM_ cells have the potential to prevent *B. pertussis* colonization in the respiratory tract. The proportion of IL-17A-secreting CD8^+^ T_RM_ cells in the LQ-aP group was significantly higher than that in AL-aP.

In summary, LQ-aP produces a high antibody, Th1, Th17, and T_RM_ response in a mouse model, providing immune protection against *B. pertussis* infection, and therefore might be used as a DTacP vaccine candidate. However, the efficacy of LQ-aP regarding diphtheria and tetanus toxin needs further verification.

## Figures and Tables

**Table 1:** Characterization of LQ-aP and AL-aP vaccines.

Adjuvant	Antigens	Particle Size (mean ± SD)	Zeta Potential (mean± SD)	Antigen Adsorption (%)
Liposome + QS-21 (LQ)	-	141.80 ± 1.04 nm	−11.57 ± 0.50 mV	n.a
	+	148.27 ± 2.25 nm	−3.39 ± 0.32 mV	n.a
Alum	-	2.61 ± 0.01 μm	22.53 ± 1.40 mV	n.a
	+	6.16 ± 0.15 μm	10.03 ± 0.56 mV	97.0

Antigens include PT, FHA, PRN, and Fim2/3. n.a, not applicable. Each sample was tested in 3 times.

## Data Availability

Data are available for scientific purposes after written request to the corresponding author.

## Abbreviations

aP	Acellular pertussis
CD	Cluster of Differentiation
CFU	Colony forming unit
CpG	Cytosine phosphoguanine
FHA	Filamentous hemagglutinin
Fim2/3	Fimbriae type 2 and type 3
IFN-γ	Interferon γ
PRN	Pertussis Pertactin
PT	Pertussis toxin
SD	Standard deviation
T_RM_	Tissue-resident memory T
wP	Whole-cell pertussis
